# Viscoelastic Testing for Coagulation Management in Vascular Surgery—A Narrative Review

**DOI:** 10.1002/hsr2.71836

**Published:** 2026-02-22

**Authors:** Alexandru Munteanu, Elena Bax

**Affiliations:** ^1^ Anaesthetic Core Trainee, Royal United Hospital Bath NHS Foundation Trust Bath UK; ^2^ Internal Medical Trainee, University Hospitals Sussex NHS Foundation Trust Brighton UK

**Keywords:** point‐of‐care devices, ROTEM, TEG, vascular surgery, viscoelastic testing

## Abstract

**Background:**

Viscoelastic testing (VET) has been increasingly used in cardiac, trauma, and hepatic surgery where it helps tackle the development of complex coagulopathies. Despite this, data on using VETs in vascular surgery remains limited to small and non‐randomised control studies. Although the improvement in vascular techniques has decreased the rate of massive haemorrhages, there remain situations where they are a serious risk, and the associated coagulopathies pose a challenge to manage.

**Aims:**

The aim of this paper is to review the current approved uses of VET and its potential translational benefit in vascular surgery.

**Methods:**

An initial PUBMED search provided the foundation for chain sampling to identify relevant papers.

**Results:**

With VET, clinicians can rapidly assess a patient's global haemostatic function thus allowing the timely, or pre‐emptive, correction of coagulopathies. Using the platelet analysis functions pre‐operatively helps guide anaesthetic technique as well as temporary discontinuation of anti‐platelet therapy, an almost ubiquitous drug among vascular patients. Intra‐operatively, the use of this point‐of‐care test, along with goal‐directed algorithms, reduces the volumes of platelets, fibrinogen, and plasma that are transfused. During recovery, using VET can help distinguish a coagulopathic bleed from one requiring surgical re‐exploration. Besides reducing the well‐established adverse effects of transfusions, from electrolyte disturbances to the more rare but severe graft‐vs‐host disease, there is a significant cost‐reducing effect.

**Conclusions:**

Although there is limited evidence of VET in vascular surgery, there seems to be a clear benefit from its use warranting further large randomised controlled trials from which standardised guidelines can be produced.

## 1 Introduction

### 1.1 Background

The aim of this review is to use chain sampling of current research to describe viscoelastic testing (VET), highlight its current perioperative uses, and describe how it may translate to uses in vascular surgery. The use of VET in vascular surgery is not yet standardised, with no national guidance dictating its use. Despite its increasing successful use in liver transplantation, cardiac surgery, and trauma, there is a paucity of research on the use of VETs in vascular surgery—limited to small cohort or retrospective studies [1]. Having recognised the clear improvement to patient outcomes, NICE Diagnostic Guidelines [2] currently recommend the use of VET in cardiac surgery. Although vascular surgery has historically been associated with large amounts of bleeding, the advent of endovascular techniques and advances in peri‐operative care has seen major haemorrhage become less problematic. Nevertheless, there are still occasions during major vascular surgery, such as ruptured abdominal aortic aneurysms or open thoracoabdominal aneurysm repairs, where management of coagulopathy and blood loss remains a challenge to anaesthetists [3]. Furthermore, in this population of patients the use of anti‐platelet therapy has become routine, which can present a clinical conundrum in their perioperative care: balancing the risk of increased bleeding if continued versus risk of thrombosis if transiently withheld. Currently, clinicians are using VETs at their own discretion, with higher prevalence in patients they assess to be high risk [4].

Quick assessment of haemostatic function, in such cases, allows coagulopathies to be treated in a timely and targeted manner reducing the amount of ongoing bleeding. Identifying a normal coagulation profile also reduces the amount of unnecessary blood component transfusion, along with its associated risks. Finally, a qualitative analysis of platelet function facilitates the decision‐making process regarding the impact of anti‐platelet therapy, its transient withdrawal and subsequent safe continuation. Taking advantage of the diverse information provided by VET, and consequently tailoring haemostatic management, not only improves patient outcomes, but can also significantly reduce hospital costs [5, 6, 7] (up to 2500 euros per patient [7]).

### 1.2 Viscoelasting Testing

VET was first described by Helmut Hartert in 1948 [8], after which thromboleastography was used in the Vietnam War to guide blood component therapy. Since then, it has been increasingly used in liver transplantation, cardiac surgery, and trauma, where complex coagulopathies are common.

VET is a point‐of‐care comprehensive analysis of global haemostatic function in real time. Transfusion algorithms can use VET's additional information and guidance to improve patient care by tailoring allogeneic blood product usage. Current standard laboratory tests (SLT) measure the initial thrombin burst in plasma, only a snapshot of a complicated and intricate system, but retain a strong correlation with detrimental outcomes among bleeding patients [6, 9]. VET provides an invaluable insight into all stages of haemostasis – from clot initiation to break down – while simultaneously reducing the time from venepuncture to results, guiding therapy in a more timely and directly applicable manner.

The UK NHS reports up to 15%‐30% of allogeneic blood transfusions in the UK NHS are inappropriate [10]. Without a tailored approach, a more liberal method of transfusing blood components is likely and is associated with a preventable rise in adverse effects ranging from simple febrile reactions to potentially fatal graft‐versus‐host‐disease or acute lung injury [11]. This has led to a paradigm shift advocating for the sparing use of blood components [12, 13]. Currently, there is only limited data of VET being used in vascular surgery. However, there is significant transferability of the application of VET from cardiac and trauma surgery.

### 1.3 TEG and ROTEM Devices

**Figure 1:**
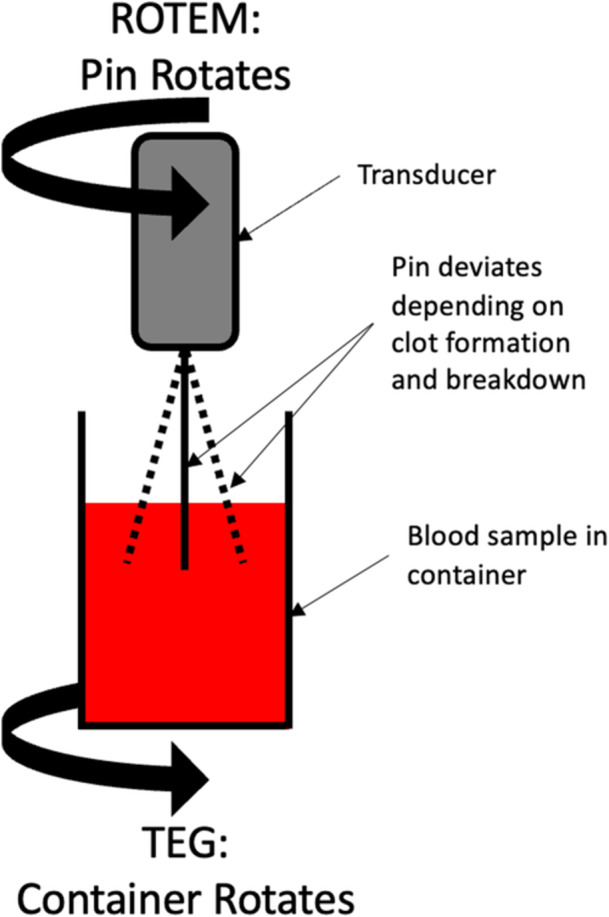
Diagram of the simplified mechanism of the original ROTEM and TEG systems.

The two most used VET systems are TEG (Haemonetics Corporation, MA, USA) and ROTEM (Haemoview Diagnostics, Brisbane, AU) devices, using different techniques, thromboelastography and thromboelastometry respectively, to achieve a similar goal. Both rely on the changes in relative movement between a pin and a cup with a blood sample caused by the developing clot (Figure 1 *)*. In TEG, torque is detected electromechanically by a transducer attached to the end of the angled pin suspended in an oscillating cup of whole blood. ROTEM relies on optomechanical detection using a mirror on the angled oscillating pin suspended in a sample of blood. The angle of the pin changes as a thrombus is formed and then broken down providing a graphical representation of all the steps of haemostasis.

The technical developments over the last 50 years have allowed these to become bed‐side tests carried out by non‐technical personnel [14]. The newest generation VET devices, TEG‐6 and ROTEM‐Sigma, have been validated to the same level as their predecessors, TEG‐5000 and ROTEM‐Delta, respectively. TEG‐6S changed the detection system – now relying on LED illumination to detect changes in the resonance of the blood sample. The updates, most notably, have transformed the ease‐of‐use of both devices, with automation of the manual handling steps needed to prepare the sample for analysis, while also increasing the number of assays that run simultaneously. The reduction in complexity and inter‐operator variability along with more comprehensive analyses are paramount for their integration into common clinical practice [15, 16].

### 1.4 Interpreting Results

**Figure 2:**
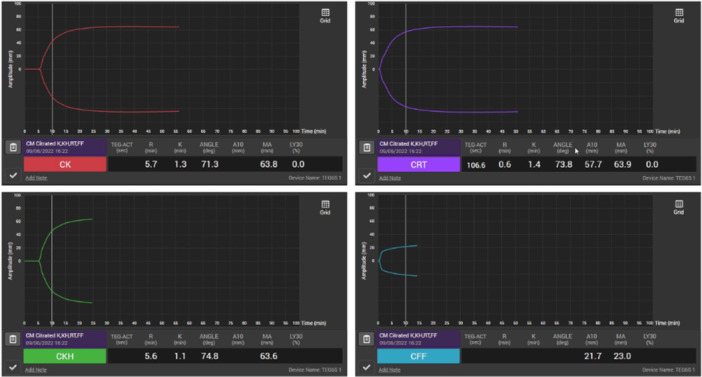
Example normal TEG‐6S results from the citrated‐kaolin CK‐TEG (left upper), citrated‐kaolin‐ tissue factor RapidTEG CRT‐TEG (right upper), citrated‐kaolin‐heparinase CKH‐TEG (left lower), and citrated‐functional fibrinogen CFF‐TEG (right lower).

The four major parameters measured by VET are clot initiation time (CIT), clot dynamics (CD), clot strength (CS), and clot breakdown (CB) (Figure 2, Table 1 *)*. CIT (R_TEG_; CT_ROTEM_), defined by the duration until any deviation from baseline, is principally a measure of coagulation factor function. CD, combining the time for amplitude to reach 20 mm (k_TEG_; CFT_ROTEM_) and the angle of the deviation (α‐angle_TEG/ROTEM_), measures the role of fibrinogen and the subsequent thrombin burst initiating the fibrinogen amplification phase. CS, identified as the maximum amplitude of the deviation (MA_TEG_; MCF_ROTEM_) strongly depends on the function and interaction of platelets and fibrinogen [17]. CB, the lysis index, percentage decrease in amplitude 30 or 60 min after reaching MA (LY30_TEG_ or LY60_TEG_; CLI30_ROTEM_ or CLI60_ROTEM_), represents the physiological, or supraphysiological, fibrinolytic activity. This is calculated slightly differently between the two devices, with the LY30_TEG_ or LY60_TEG_ representing the percentage decrease in amplitude 30 or 60 min after achieving MA [18] while the CLI30_ROTEM_ or CLI60_ROTEM_ representing the ratio between the amplitude at 30 or 60 min after CT [19]. Using these principal parameters, the devices can calculate and derive further indices, most commonly Clotting Index, representing the continuous global assessment of clot formation [20]. Although both TEG and ROTEM measure the same parameters, the techniques differ enough to make results nontransferable between the devices.

**Table 1:** VET parameters with the phase of haemostasis they described and associated principal variable.

Abbreviation	TEG	ROTEM	Description	Determining variable
CI	R	CIT	Clot initiation	Clotting factors
CD	k	CFT	Amplification phase	Fibrinogen
	α‐angle		Thrombin burst	Thrombin
CS	MA	MCF	Clot strength	Platelets Fibrinogen
CB	LY30/LY60	CLI30/60	Fibrinolysis	Plasmin: anti‐plasmin ratio

Each device has several different assays that can be used to unroot the cause of the coagulopathy for targeted treatment to be administered. The main modules used rely on contact (CK‐TEG; INTEM) and tissue factor (TF) activation (EXTEM; RapidTEG) to quantify the intrinsic and extrinsic coagulation pathways, respectively. Functional Fibrinogen (CFF‐TEG) and FIBTEM are assays which inhibit platelet aggregation to isolate the haemostatic function of fibrinogen. However, these indirect methods of measuring fibrinogen assume all other coagulation parameters to be within normal range [9], which may not be the case in this cohort of patients. Other assays can eliminate the residual effects of heparin using heparinase (CKH‐TEG; HEPTEM) and identify fibrinolysis (APTEM). RapidTEG (CRT‐TEG) is especially useful in patients with known coagulation factor dysfunction, in order to rapidly assess CS by shortening the CIT.

### 1.5 Platelet Function Testing (PFT)

With cardiovascular disease as the leading cause of death in the developed world, there is a continuously increasing population taking anti‐platelet therapy. The well‐established interpersonal variability of response [9] makes it informative to analyse a patient's individual response status. Both TEG and ROTEM have developed analysers for this purpose. ROTEM‐Platelets relies on aggregometry, adapted from the widely used multiple electrode aggregometry, whereas TEG‐Platelet Mapping (TEG‐PM) calculates platelet inhibition as a proportion of maximum clot potential.

## 2 VET in Vascular Surgery

### 2.1 Preoperative

Bleeding history, including a physical examination, remains the only evidence‐based method of identifying patients at higher risk of bleeding [21]. SLT can be used in conjunction to help guide preoperative preparation (e.g. reversal of warfarin, and identification of rare bleeding disorders such as haemophilia or von Willebrands disease) [21]. While aspirin can be continued without adverse effects on bleeding [14], it is currently recommended that the anti‐platelet agents prasugrel, clopidogrel, and ticagrelor should be discontinued 7, 5, and 3 days preoperatively, respectively [22]. With a large proportion of vascular surgery patients requiring dual‐/mono‐anti‐platelet therapy, a fine balance between thrombotic complications and bleeding risks must be struck. Often the bleeding risks are over‐estimated, whilst thrombotic ones overlooked, leading to inappropriate withholding of anti‐platelets. This is especially crucial in patients undergoing carotid endarterectomies and peripheral endovascular interventions whose risk of stroke and coronary artery thrombosis are high. VET‐based platelet function testing has a potential use in risk‐stratifying patients receiving anti‐platelet therapy.

In the subgroup of patients where the risk of stopping anti‐platelets proves to be too high, or when these patients present for emergency surgery, PFT can be used preoperatively to assess a patient's level of platelet inhibition. Among off‐pump cardiac surgery patients, evidence supports using 70% ADP‐platelet receptor inhibition (ADP‐PRI) as the cut‐off predicting increased transfusion requirements [23]. Further investigation into non‐cardiac surgery suggests that a lower threshold, 34% ADP‐PRI, predicts adverse outcomes and could be used to in the consideration of proceeding with or cancelling surgery [24]. With no correlation between length of discontinuation platelet inhibition [24], PFT aids clinicians in the timing of surgery, discontinuation of anti‐platelet therapy, and anticipating blood requirements.

Stratifying patients by platelet inhibition can also be useful in patients in whom the avoidance of general anaesthesia is desirable due to comorbid status – where a regional anaesthesia technique may be considered as an alternative. Platelet inhibition would not only guide haemostatic preparations, but also direct the decision of the anaesthetic technique used. However, it should be noted that large prospective studies of neuraxial blockade in patients taking anti‐platelet medication are unlikely to be conducted due to the number of patients required to power such a study.

Other VET parameters can also be used preoperatively to risk stratify patients and guide haemostatic management. Preoperative CS has been shown to correlate with future blood requirements, with TEG‐MA < 42.5 mm having a high specificity and sensitivity in predicting increased post‐operative bleeding (sensitivity 78%; specificity 86%) [25]. PFT, in conjunction with bleeding history, SLT, and preoperative VET, can help profile high haemorrhage‐risk patients. However, the increasing use of direct oral anticoagulants, inhibiting clotting factors, requires a better system to monitor their effects. EXTEM‐CT was the only parameter that was reliably prolonged with Dabigatran, Apixiban, and Rivaroxaban. Other studies showed that ROTEM [26] poorly detects DOACs while TEG‐6S has a promisingly high sensitivity and specificity [27]. Although an encouraging potential use of VET, further research is required before they can be reliably used in common clinical practice.

### 2.2 Intraoperative

#### 2.2.1 Transfusion Protocols

Damage Control Resuscitation (DCR), relying on blood component ratios, are frequently used in emergency department. By ensuring the prompt, balanced administration of blood products, DCR helps prevent the development of the lethal triad: coagulopathy, hypothermia, and acidosis. Notably, turnaround for SLTs are known to close to an hour [28], consuming precious time for patients in critical situations. If unable to identify the specific cause of the coagulopathy, more likely if the SLT results are not ready, DCR can lead to high volume transfusions of all blood products to ‘cover all bases’. Consequentially, a substantial number of patients receive inappropriate transfusions associated with morbidity and mortality relating to complications including acute lung injury, transfusion‐associated circulatory overload, and GVHD [29], or more commonly citrate‐toxicity and dyselectrolytaemia. The costs of transfusion‐associated adverse effects averages at 2,800 euros per patient on the healthcare system alone, not accounting the social costs associated to lost working days and deaths [30].

In order to tackle inappropriate transfusions, protocols relying on both clinical judgement and laboratory tests were developed in the surgical department to achieve a targeted approach to patient management. Including VET parameters, with results available within minutes, even just CS, on top of SLT is enough to significantly reduce the number of patients receiving FFP and platelet transfusion. This reduction is not limited just to the intra‐operative period but is seen to extend into the post‐operative phase. Using more VET parameters to guide therapy reduces PRBCs as well [31]. Incorporating VET into DCR algorithms not only optimises management and reduces over‐transfusion, but it also helps reduce inter‐clinician variability as well as aiding training.

The 2021 ITACTIC trial compared VET data‐driven algorithms [32], developed to identify trauma‐induced coagulopathy, to SLT transfusion algorithms, in trauma patients. This international multi‐centre RCT showed no significant difference in primary outcomes, proportion of patients alive and free of massive transfusion at 24 h. There was, however, a reduction in mortality and major transfusions in the pre‐defined traumatic brain injury subgroup analysis [33]. This again suggests there are potential groups of patients requiring major transfusions, such as high‐risk vascular surgery patients, who may benefit from the use of VET.

#### 2.2.2 Heparinase Assays: Extracorporeal Circulation (ECC) in Cardio‐Pulmonary Bypass

It is well established that the use of ECC causes changes in the physiological haemostatic processes. From the heparin administered to the synthetic tubes indiscriminately activating and consuming clotting factors, to the ECC sequestering platelets, the end result remains a multifactorial coagulopathy. In order to reduce allogeneic blood transfusion in such circumstances, it is vital to identify the cause, or causes, of the coagulopathy. The heparinase assays (HEPTEM; CKH‐TEG) play a vital role in these patients during the rewarming phase as well as post‐ECC.

During the rewarming phase, it is vital to identify potential coagulopathies that will manifest once ECC is discontinued. The heparin circulating in the patient renders all SLT null. However, VET assays containing heparinase aim to neutralise the heparin and identify any deficiencies in clotting factors, fibrinogen, or platelets, allowing pre‐emptive targeted transfusion. This helps reduce haemorrhages and the consequential FFP and PRBC requirements by 50% [9] and 87.5% [34], respectively, without increasing post‐operative bleeding [34]. There remains a risk, however, that the dose of heparinase is not sufficient to fully inactivate the sample and thus affecting interpretability of results.

Post‐ECC, following heparin neutralisation using protamine, a prolonged CIT indicates clotting factor dysfunction, but this is confounded if there is residual heparin. This can be demonstrated by the resolution of the prolonged CIT using a heparinase assay, suggesting the patient requires further protamine administration. An unresolving prolonged CIT on the heparinase trace suggests a coagulopathy requiring clotting factor replacement using FFP.

#### 2.2.3 Platelets and Fibrinogen

Thrombocytopenia and hypofibrinogenaemia can be identified by an abnormal CS. Normal platelet function, and subsequent thrombus formation, relies on adequate fibrinogen levels. Thus, Functional Fibrinogen or FIBTEM is recommended in the first instance. Currently, due to the multifactorial nature of the coagulopathies identified in haemorrhagic patients, target fibrinogen levels have been raised from 80 to 100 mg/dL to 150–200 mg/dL [35]. FIBTEM amplitude at 10 min (FIBTEM‐A_10_) of 8–10 mm has been validated to correspond to the desired fibrinogen concentration of 150–200 mg/dL. FIBTEM‐A_10_ < 5 mm has often been described in trauma‐induced coagulopathies and must be corrected with fibrinogen replacement before assessing platelets using EXTEM‐A_10_, validated to trigger platelet transfusion if below 40 mm. Not only does a FIBTEM‐A_10_ dictate immediate management, but it is an early predictor of post‐operative bleeding [36]. The FIBTEM assay contains heparinase, facilitating its use during the rewarming phase of ECC [29] so allowing timely restoration of fibrinogen levels, which can prevent the development of the aforementioned lethal triad.

#### 2.2.4 Anti‐Fibrinolytic Therapy

Local fibrinolysis, by action of tissue plasminogen, is a normal haemostatic function allowing the return of normal blood flow. However, inflammation and haemodilution dysregulates the fine balance of pro‐fibrinolytic and anti‐fibrinolytic signals. High concentrations of plasminogen activator inhibitor 1 and α_2_ antiplasmin limit fibrinolysis to the local milieu. However, haemodilution and ECC consumption of anti‐fibrinolytic factors increases susceptibility to hyperfibrinolysis [11, 36]. With CB elevated in fibrinolysis, clot index can distinguish physiological primary fibrinolysis, where it is decreased, from pathological secondary fibrinolysis with an increased clot index [37].

Although VET seems to be useful in assessing clot breakdown, it is limited to detecting only severe fibrinolysis, insensitive to mild‐moderate levels [36]. Promising research suggests the addition of different concentrations of tissue plasminogen activator improves the ability to identify different grades of fibrinolysis [38]. Currently ROTEM has developed an assay to help elucidate unclear fibrinolytic traces on the EXTEM by the addition of aprotinin in APTEM. The resolution of fibrinolysis on APTEM confirms pathological clot breakdown [29] requiring treatment with tranexamic acid (TXA), or in some rarer instances ε‐aminocaproic acid. However, VET's use in guiding intra‐operative anti‐fibrinolytic therapy is further limited by evidence from the CRASH‐2 trial, showing ubiquitous early administration of TXA to trauma patients at risk of, or already, bleeding reduces mortality [39]; and the haemorrhage‐reducing effects of TXA are extended to the postoperative period [36]. Using VET in these circumstances to ascertain hyperfibrinolysis would delay TXA administration and potentially cause detrimental harm to patients [9].

#### 2.2.5 Evidence in Vascular Surgery

**Figure 3:**
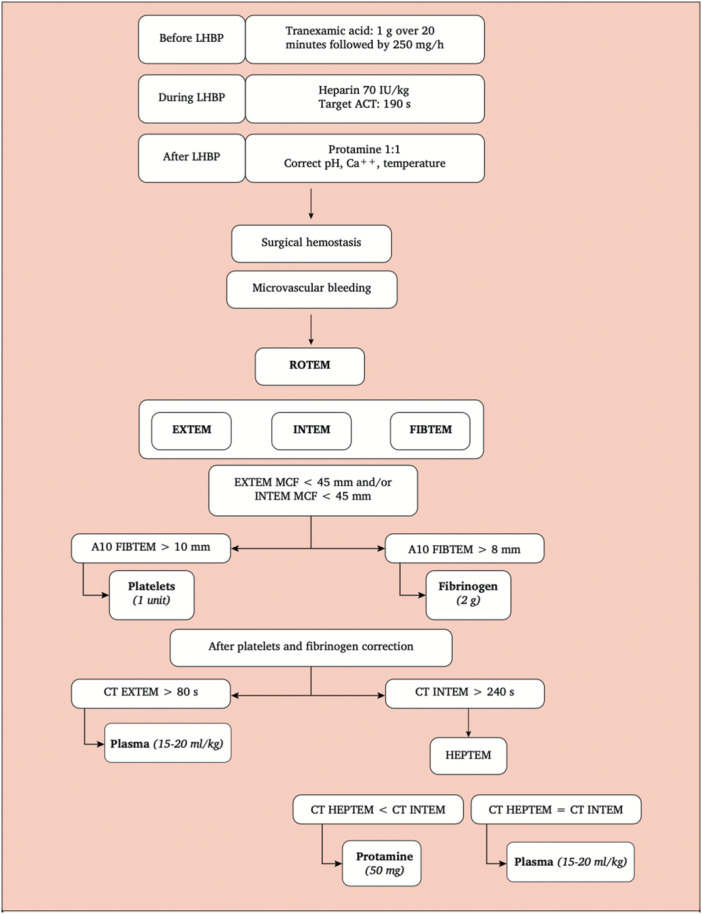
Example of ROTEM‐guided transfusion protocol used by the San Raffaele Scientific Institute (Milan, Italy) in open thoraco‐abdominal aortic aneurysm repairs. [43] (LHBP = Left Heart Bypass Pump; ACT = Activated Clotting Time; MCF = Maximum Clot Firmness (CS); CT = Clotting Time; Ca = Calcium).

The limited, yet promising, data regarding the use of VET in vascular surgery shows a reduction in amount of blood transfused per patient [40]. Although not preventing the need for transfusion, VET facilitates a targeted and rapid correction of coagulopathies. Intra‐operative haemostatic management guided by VET not only reduces the immediate requirement of all allogeneic blood products [41, 42], but also the post‐operative requirements. Furthermore, there is an accompanied reduction in post‐operative haemorrhagic and thrombotic complications [7]. The beneficial effects of VET in vascular surgery are not limited to elective patients, who are well controlled and pre‐assessed, but also to emergency surgeries at higher risk of requiring massive transfusions [42]. A recent 2019 retrospective study [43] of 546 patients undergoing elective, urgent, and emergency open thoraco‐abdominal aortic aneurysm repair at San Raffaele Scientific Institute, a national referral centre in Milan, Italy, compared SLTs and ROTEM driven transfusion algorithms (Figure 3). They noted a reduction in transfused blood products with a significant reduction in pulmonary complications and hospital‐associated costs in those guided by ROTEM. The results are promising however considering the retrospective and observational nature of the study highlights the need of large‐scale prospective RCTs.

### 2.3 Post‐Operative

#### 2.3.1 Post‐Operative Bleeding

One of the predominant post‐operative complications in major vascular surgery is reoperation due to bleeding [37]. For patients showing signs of bleeding, it is crucial to distinguish surgical bleeding from coagulopathy. VET allows a rapid and complete analysis of haemostasis thus identifying any room for treatment with blood products prior to, or preventing the need for, surgical re‐exploration. Strong evidence from multiple RCTs and systematic reviews demonstrates the use of VET is associated with lower rates of re‐exploration [9, 17, 44, 45]. However, other systematic reviews, including a Cochrane Review, have failed to elucidate such a link and suggest VET‐guidance is limited to reducing transfusion requirements [46, 47].

#### 2.3.2 Post‐Operative Thrombosis

Post‐operative thromboembolic complications can be found in up to 10% of vascular patients, conferring a four‐fold increase in mortality [48]. Although there are well established risk factors, such as duration of procedure directly correlating with an increased risk [49], it remains difficult to accurately predict. The role of VET in predicting hypercoagulability, remains debated. Although retrospective analysis of patients suffering from thrombotic complications demonstrated increased CS and shortened CIT, prospective interpretation of these parameters is complicated due to the supraphysiological procoagulant stimuli used in the assays [11]. Currently, with no validated parameter or threshold, the predictive accuracy of VET remains highly variable, but CS least so [50]. A study of 240 patients showed that TEG‐MA > 68 mm was significantly related to increased incidence of thrombotic complications, including myocardial infarctions [37]. Using VET to confirm post‐operative patients to be non‐coagulopathic also facilitates a timely initiation of prophylactic low‐molecular weight heparin as well as restarting any anticoagulants. Even with VET‐based early identification and reduction of thromboembolic events, no overall reduction in mortality has been demonstrated [5].

### 2.4 Limitations of VET

Aside from the numerous benefits of using VET, there remain limitations that need to be considered. One such limitation, of technique itself, is the artificial way in which it assesses clotting using a low shear‐stress environment to predict coagulopathies developing in the high shear‐stress arterial system [11]. The most contested limitation is the inability so far for VET to demonstrate any reduction in mortality [47]. However, it is debated that that is not the goal, rather being designed to affect transfusion rates to improve patient care and save resources [5]. Although there is limited evidence of VET in vascular surgery, there are several similarities with cardiac and trauma surgery, especially relating to complex multifactorial coagulopathies, effects dilution due to ECC, and the different transfusion algorithms. This suggests similarly beneficial effects in vascular surgery.

### 2.5 Research Uses

Novel research looking at the increasingly important role that fibrinogen has in perioperative haemostasis uses VET. ROTEM was used to determine that fibrinogen concentrate reduced allogeneic blood requirements in patients undergoing thoraco‐abdominal aortic aneurysm repairs when compared to standard FFP [51]. The TEG‐CABG trial used VET, along with MEA, to assess hypercoagulability post‐CABG. Conclusions from this RCT indicated that DAPT, although reducing mortality and MI rates, did not reduce venous graft occlusion [52]. Two studies conducted by Rahe‐Meyer et al., using FIBTEM‐MCF to guide fibrinogen replacement therapy with concentrate or FFP, showed that although fibrinogen concentrate significantly reduced allogeneic blood product transfusion in major aortic replacement surgery [53], it increased transfusion rates in complex cardiac surgery [54]. Further research with VET optimising fibrinogen therapy remains to be conducted.

## 3 Conclusion

VET presents a continuously developing piece of equipment, with clear potential to have a significant positive impact on patient care in vascular surgery. Multiple national guidelines [55, 56], including NICE Diagnostic Guidance, currently support the use of VET in cardiac surgery, with clear improvements in outcomes, such as time spent on critical care, in hospital, as well as reducing complications, and costs brought on by excessive use of allogeneic blood components. With increasing evidence from the transferability of these results to vascular surgical patients, there is a clear benefit of its use in patients at high risk of excessive bleeding. In contrast to its clear beneficial trend, current evidence remains sparse and weak, limited only to surrogate outcomes [46] with large prospective randomised controlled trials and standardised protocols required for clinical outcomes.

## Author Contributions

**Alexandru Munteanu:** writing – review and editing, writing – original draft. **Elena Bax:** writing – review and editing.

## Funding

The authors received no specific funding for this work.

## Conflicts of Interest

The authors declare no conflicts of interest.

## 1 Transparency Statement

The lead author Alexandru Munteanu affirms that this manuscript is an honest, accurate, and transparent account of the study being reported; that no important aspects of the study have been omitted; and that any discrepancies from the study as planned (and, if relevant, registered) have been explained.

## Data Availability

Data sharing not applicable to this article as no datasets were generated or analysed during the current study.
